# Impact of Covid-19 on the restless legs syndrome

**DOI:** 10.5935/1984-0063.20200031

**Published:** 2020

**Authors:** Beatriz Franco, Milca Abda Morais, Alessandro Spencer de Souza Holanda, Mauro Manconi, Marco Túlio de Mello, Andrea Maculano Esteves

**Affiliations:** 1 Universidade Estadual de Campinas, Faculdade de Educação Física - Campinas - Sao Paulo - Brazil.; 2 Universidade Estadual de Campinas, Faculdade de Ciências Aplicadas - Limeira - Sao Paulo - Brazil.; 3 Civic Hospital of Lugano (EOC), Sleep and Epilepsy Center, Neurocenter of Southern Switzerland - Lugano - Ticino - Switzerland.; 4 Universidade Federal de Minas Gerais, Escola de Educação Física, Fisioterapia e Terapia Ocupacional - Belo Horizonte - Minas Gerais - Brazil.

**Keywords:** Anxiety, Coronavirus, Depression, Exercise, Heart Diseases, Sleep-Related Movement Disorder

## Abstract

The COVID-19 pandemic is strongly impacting global health and mental health, and has caused routine changes in much of the world. All of these changes can have physical and mental consequences for the population. Metabolic changes, mood disorders and their pharmacotherapy, such as the use of antidepressants can increase the chances of developing sleep disorders, such as Restless Legs Syndrome (RLS). As a way to avoid immunosuppression and the greater risk of contamination of COVID-19, it is recommended to perform exercise in light or moderate intensity. This is the ﬁrst paper to reﬂect a possible impact of social distancing on RLS, and how it can affect the incidence of mood disorders and metabolic changes in this syndrome, as well as the quality of life of the population. We suggest that regular exercise at light or moderate intensity during home conﬁnement may be effective strategies to minimize the changes caused by the COVID-19 pandemic.

## COVID-19 AND RESTLESS LEGS SYNDROME

In December 2019, the COVID-19 virus was officially reported in China, through the human contagion of the SARS-CoV-2 virus, which is genetically similar to SARS- CoV-1. Very quickly, the virus spread to many other countries across all continents^1^.

The main symptoms firstly reported to be associated with COVID-19 are sore throat, dry cough, fever (mild symptoms), loss of olfactory and taste, pneumonia, pulmonary edema, and acute respiratory distress syndrome (severe cases)^2^. Symptoms are similar across ages; however, they tend to be more severe in old age. Regardless of age, the symptoms, as well as prognosis, can be more severe in the presence of comorbidities such as cardiovascular disease, diabetes and hypertension^3^.

In February 2020, the World Health Organization declared the COVID-19 as a threat to public health worldwide. To date, there are no validated treatments or vaccines, and thousands of severe cases crowd intensive care units and die every day worldwide. Until 18th May 2020, the world has more than 4,5 million confirmed cases and more than 310 thousand deaths from COVID-19^4^.

To prevent collapse of the health systems, to reduce the lethal burden of the disease and to limit the circulation of the virus, the WHO suggested a lockdown strategy and some behavioral rules like washing hands frequently, keeping social distance and wearing a mask. Nowadays, the entire world is facing one of the hardest challenges in public health of the last century, with most of population at home confinement for weeks or months.

Studies have shown that physical inactivity and a lack of regular social interaction in home confinement affect the physiological health of body and mind^5,6^. Isolation due to in-home has caused changes in mood, metabolism and sleep, and an increased sedentary lifestyle in people.

Metabolic and mood disorders are closely related to changes in sleep, affecting as an example the Restless Legs Syndrome (RLS), which is a sensory-motor disorder that causes difficulty in initiating and maintaining sleep^7^ and has a close relationship with metabolic and mood changes^8,9^.

RLS is characterized by discomfort in the limbs (especially in the legs) at night, before starting to sleep. The symptom follows by urge to move and inability to falling and maintain sleep^10^. Although uncertain, dopaminergic dysfunction is the most sustain pathogenetic hypothesis until now^11^. Associated with RLS (80% of cases), periodic leg movements might contribute to sleep fragmentation^10^. At the end, RLS affects sleep efficiency and quality of life, resulting in a lack of energy during the day, mood swings and difficulty socializing, and carrying home activities^7^.

Based on this, the behavioral changes caused by COVID-19 might worsen or trigger symptoms of RLS in predisposed people. This paper is the first to review the possible effect of home confinement on RLS in the world pandemic of COVID-19.

## MOOD AND RLS

Home confinement caused by the new coronavirus pandemic (COVID-19) has caused financial, psychological and emotional problems. Consequently, there may be an increase in mood disorders, such as higher levels of anxiety and depression^6^. Besides, studies have also shown that confinement is associated with increased psychological distress and higher levels of stress^6,12^.

Most people are going through significant changes in their daily routines, living with uncertainty, insecurity about health and economy, and concern about the situation and duration of the pandemic. Due to the school closure, working parents need to reconcile their work schedule with their children’s caregiving, this in addition to usual domestic administration. Entrepreneurs and those with small businesses or start-ups, or even those who work in sectors that are currently closed, such as entertainment, hospitality, bars and restaurants, or other enterprises, are probably suffering from stress and anxiety about the continuity of job and financial security^13^.

Besides that, confinement can create additional stress due to people’s inability to engage in pleasurable activities, such as visiting friends and family, shopping, participating in cultural and sporting events and attending bars or restaurants. Spending more time with the family can also induce stress, especially in situations where there are pre-existing family difficulties. All of these changes can contribute to the increase in anxiety symptoms and, consequently, reduce the quality of sleep^13^. In addition to anxiety, depression symptoms are directly related to sleep disorders, and just like mood disorders can lead to sleep disorders, the reverse is also true^14^.

Studies demonstrate an association between RLS and mood disorders^9,15^. Sleep disruption due to RLS symptoms hurts mood, contributing to higher rates of depression and anxiety^15^.

The linkage between RLS and psychiatric disorders is still unclear, however, some hypothesis have been raised to explain this association. One of them is that sleep architecture is negatively affected by both RLS and mood disorders^9,16^. Many antidepressants and antipsychotics can worsen sensory symptoms of RLS, sleep quality and pre-existing psychiatric disorders^15^. Another hypothesis is that RLS and mood disorders may share similar pathophysiological mechanism and cerebral networks. For instance, dopaminergic dysfunction is implicated in the pathophysiology of both RLS and depression^9,11,17^.

In this sense, considering this association between RLS and mood disorders, and the consequent impact on the quality of life, it is of great importance to carry out strategies to improve the quality of sleep, to prevent and reduce the symptoms of anxiety and depression during the confinement period caused by COVID-19.

## METABOLIC CHANGES AND RLS

The rapid spread, the absence of treatment, and the increase of contagion and deaths due to the COVID-19 pandemic made a large part of the world take social distance as protection. Prior to COVID-19, people practiced physical activity to stay healthy or to improve some comorbidities such as diabetes mellitus 2 (DM2), hypertension and cardiovascular diseases^18^. With the home confinement, people’s physical activity level decreased, as expected. A study carried out during the pandemic for an electronics company showed that the physical activity of the population can be reduced in 38%^19^, suggesting a similar decline to other confined conditions, where there is an increased physical inactivity^20^. The damage that physical inactivity can cause in the population, such as increased incidence of cardiovascular disease, hypertension and diabetes, is already well established in the literature^5^.

In a review paper, Walters and Rye^8^ showed that epidemiological studies suggest a possible relationship between cardiovascular disease and hypertension with RLS. Also, high prevalence of RLS was reported in type 2 diabetics^21^.

Literature demonstrated that patients with heart disease and hypertension presented a higher prevalence of RLS as opposed to healthy people^22^. It is not clear by which mechanisms this relationship occurs, however the hypotheses discussed are: dopaminergic alteration (reduction of dopaminergic receptors) caused by increased sympathetic activity due to changes in the heart (physiological stress conditions)^23^; vascular changes in the central or peripheral nervous system; and medication use^22^.

Studies with DM2 patients also showed an increase prevalence of RLS in these patients^21^. This relationship can be related to changes in the dopaminergic system (dopamine reduction), caused by DM2^24^.

A recent study showed that patients with moderate and severe symptoms of RLS have sympathetic hyperactivity^25^. The increase in sympathetic activity projects a fight or flight reaction and an increase in cortisol, causing a waking state, metabolic excitation (increase heart rate and blood pressure) and excitation of movements. The increase in sympathetic arousal that occurs in patients with RLS is related to the reduction and fragmentation of sleep, and with constancy, they are associated with health risks, such as hypertension and heart disease^25,26^. RLS can cause an increase in the incidence of hypertension and heart disease. Also, RLS related sleep disruption favors other metabolic consequences, such as type 2 diabetes and obesity^27^.

Thus, the reduction of physical activity caused by confinement at home may cause metabolic changes such as hypertension, diabetes and cardiovascular diseases, which may cause RLS. RLS can worsen the quality of life and sleep of people, who probably already have several physical and mental changes due to the pandemic. In addition, for those who already have RLS, the reduction of physical activity can trigger the development of comorbidities that harm health, causing greater consequences in life. Therefore, at this moment it is essential to create strategies for the practice of physical activities to mitigate the damage.

## EXERCISE AND RLS

The practice of exercise is an effective therapy in the fight against chronic diseases, metabolic syndromes, and reduction or treatment of diseases related to sleep disorders^28,29^. Guidelines and recommendations for the practice of physical activity have always been strongly disseminated and applied in different clinical health conditions^30^.

In the current context in which the COVID-19 pandemic lives, maintaining physical activity is of fundamental importance, to minimize the consequences of quarantine and reduce the risk of contracting the virus^31^. Maintaining levels of physical activity practices and a healthy lifestyle during the quarantine period are extremely important for the health of the population and especially for those with health-related risk factors^32^.

Exercise therapy associated with sleep is related to mitigating the adverse effects of sleep disorders, including RLS^29^. In this syndrome, the results of exercise can be acute and chronic, with a focus on reducing the severity of its symptoms^32,33^. The main effect of exercise on RLS is the improvement in sleep quality, sleep efficiency, the percentage of REM sleep, and the reduction in wake after sleep onset (WASO), factors that are consequently associated with improved physical and mental health and reduced mortality^33,34^. The planned moderate exercise with the correct prescription prioritizing aerobic options due to the release of beta-endorphins and neurotransmitters, such as dopamine^33^ can be ideal strategy to minimize the RLS symptoms.

Besides, to improve the symptoms of RLS, one of the main positive effects of exercise are the beneficial metabolic changes caused acutely and chronically in the patient’s body^35^. Having similar effects, the essential function in the treatment and control of comorbidities such as cardiovascular diseases and diabetes^36^, conditions considered risk factors associated with COVID-19^3^.

In addition to these, another effect of exercise is the improvement in neurobiological aspects of mental health^37^. Exercise can be an effective adjunctive intervention for depression^38^, often together with psychotherapy and/or antidepressant medication.

There are numerous possibilities for physical activity; however, during the COVID-19 pandemic it is necessary to respect the quarantine. It is possible to perform several exercises at home^39^. Recommended practice of exercise is essential and should be performed in light or moderate-intensity since high intensity can suppress immunity and become a window for contamination for the COVID-19^40^. This intensity exercise profile having beneficial effects on metabolic and RLS aspects^33^.

Therefore, it is suggested to realization exercises in this quarantine period, according to previously published studies^31,39^. Resistance exercises can be done through callisthenic and aerobic modalities through basic activities such as walking and dancing at home^31^. In addition to practices such as yoga, jump ropes, weightlifting exercises, on top of the activities of daily living^39^.

## CONCLUSIONS AND RECOMMENDATIONS

Considering the impact of COVID-19 on the sleep quality, some recommendations are essential to prevent or mitigate the changes caused by the pandemic, among them maintaining a regular sleep schedule and a quiet environment, as well as reducing stress at night (see the study for more details)^13^.

The safe practice of daily physical activity and regular PE is a way to minimize the effects of confinement at home, as well as the possible symptoms of RLS, also effective in improving cardiovascular diseases, hypertension, diabetes and mood fluctuations. Finally, exercise is considered a treatment to reduce the symptoms of RLS, enhancing the quality of sleep.

Therefore, we believe that sleep hygiene recommendations associated a regular moderate exercise, with the correct prescription, can be ideal strategies to minimize the changes caused in the pandemic caused by COVID-19.

We suggest consulting websites with recommendations for the practice of exercise during the home confinement:

- http://www.euro.who.int/en/health-topics/health-emergencies/coronavirus-covid-19/technical-guidance/stay-physically-active-during-self-quarantine
- https://www.exerciseismedicine.org/support_page.php/stories/?b=892
- https://www.heart.org/en/healthy-living/fitness/getting-active/create-a-circuit- home-workout
